# Metabolomics of Ramadan fasting and associated risk of chronic diseases

**DOI:** 10.1016/j.ajcnut.2024.01.019

**Published:** 2024-02-01

**Authors:** Rami Al-Jafar, Rui Climaco Pinto, Paul Elliott, Konstantinos K Tsilidis, Abbas Dehghan

**Affiliations:** 1 Department of Epidemiology and Biostatistics, School of Public Health, Imperial College London, London, United Kingdom; 2 Department of Data Services, Lean Business Services, Riyadh, Saudi Arabia; 3 Dementia Research Institute at Imperial College London, London, United Kingdom; 4 National Institute for Health Research Imperial College Biomedical Research Centre, Imperial College London, London, United Kingdom; 5 Department of Hygiene and Epidemiology, University of Ioannina School of Medicine, Ioannina, Greece; 6 MRC-PHE Centre for Environment and Health, School of Public Health, Imperial College London, London, United Kingdom

**Keywords:** intermittent fasting, lifestyle, metabolic biomarkers, chronic diseases, Ramadan

## Abstract

**Background:**

The dramatic change in lifestyle associated with Ramadan fasting raises questions about its effect on metabolism and health. Metabolites, as the end product of metabolism, are excellent candidates to be studied in this regard.

**Objective:**

This study aims to investigate the effect of Ramadan fasting on the metabolic profile and risk of chronic diseases.

**Methods:**

The London Ramadan study (LORANS) is an observational study in which 2 blood samples were collected from 72 participants a few days before and after the fasting month of Ramadan. We conducted metabolomic profiling using nuclear magnetic resonance spectroscopy to assess the change in individual metabolites from before to after Ramadan. Also, we generated metabolic scores (scaled from 0 to 100) for 7 chronic diseases in the UK Biobank and assessed the association of Ramadan fasting with these scores in LORANS.

**Results:**

Of the 72 participants, 35 were male (48.6%); the mean (± standard deviation) age was 45.7 (±16) y. Ramadan fasting was associated with changes in 14 metabolites (1 inflammation marker, 1 amino acid, 2 glycolysis-related metabolites, 2 ketone bodies, 2 triglyceride, and 6 lipoprotein subclasses), independent of changes in body composition. Using data from 117,981 participants in the UK Biobank, we generated metabolic scores for diabetes, hypertension, coronary artery disease, renal failure, colorectal cancer, breast cancer, and lung cancer. The metabolic scores for lung cancer, colorectal cancer, and breast cancer were lower after Ramadan in LORANS (−4.74, 9.6%, 95% confidence interval −6.56, −2.91, *P* < 0.001), (−1.09, −2.4%, −1.69, −0. 50, *P* < 0.001), and (−0.48, −1.1%, −0. 81, −0.15, *P* = 0.006), respectively.

**Conclusions:**

Ramadan fasting is associated with short-term favorable changes in the metabolic profile concerning risk of some chronic diseases. These findings should be further investigated in future, larger studies of longer follow-up with clinical outcomes.

## Introduction

Fasting is practiced by a large number of people for a variety of reasons, including weight loss and improving general health. Time-restricted fasting in particular has become popular, but comprehensive studies on the effects of such diets are lacking. Ramadan (religious) fasting is similar to time-restricted fasting, practiced by hundreds of millions of Muslims around the world [1], providing a “natural experiment” to study the potential metabolic effects of time-restricted fasting.

Metabolic changes that follow fasting in the first couple of hours include glycogenolysis (consuming the glycogen stores), followed by an adaptive phase after a more extended period of fasting (>8 h), where the insufficiency/absence of the primary fuel (glucose) is compensated for by converting glucogenic amino acids into glucose (gluconeogenesis) and converting ketogenic amino acids and fatty acids into ketones (ketogenesis) [2,3]. However, the longer-term metabolic effects of fasting and its subsequent effects on health remain to be elucidated.

The metabolic effects of fasting on different systems can be studied using various methods within the global -omics umbrella. Metabolomics is an approach where a wide range of small molecules in biological specimens are analyzed [4]. A previous study found that prolonged fasting significantly altered 166 metabolites [5], with the metabolic profile being associated with some chronic diseases [6,7]. We hypothesize that Ramadan fasting is modulating the metabolic profile that may have an effect on risk of chronic disease in the long term.

So far, only 2 small studies were conducted to explore the metabolic effects of Ramadan fasting. The first is a pilot study of Ramadan fasting using metabolomics involving measurements of 186 metabolites collected on the 7th and 26th day of Ramadan in 11 healthy males, with significant changes observed for 20 metabolites [8]. The second study used mass spectrometry to investigate the metabolomics changes in 25 individuals with overweight and obesity after Ramadan fasting [9].

Here, we report the metabolomics changes after Ramadan fasting using data and samples from the London Ramadan study (LORANS). Moreover, to speculate on the clinical impact of fasting, we built metabolite risk scores for several chronic diseases using the UK Biobank data and evaluated whether the metabolite risk score of any of these diseases was significantly modified after 1 mo of Ramadan fasting.

## Methods

### London Ramadan study

LORANS is an observational study conducted in 2019 in London, United Kingdom. Those aged 18 y and above who were planning to fast for >20 d of Ramadan were eligible for the study, with data collection carried out in clinics set up in 5 mosques in London. The study included 2 visits: one before the fasting month of Ramadan and the second within 8–12 d after the end of Ramadan. We excluded pregnant females and those who were unwilling to attend the second visit. Before Ramadan, we collected blood samples from 140 individuals of whom 78 (55.7%) participants attended the second visit and gave blood samples after Ramadan. We collected 2 EDTA blood samples (9 mL) from each participant per visit, which were transported in a thermoporter to a bio-repository at Charing Cross Hospital for processing and long-term storage at −80°C. We excluded 6 individuals whom the first visit sample was not processed because of a delay in transporting the samples for processing, leaving 72 participants with blood samples before and after Ramadan (**Supplemental** Figure 1). Supplemental Table 1 compares the study sample’s demographics (*n* = 72) to those of people not attending the second visit (*n* = 68); the 2 groups were similar except for age. In addition to blood samples, we collected socioeconomic and lifestyle data using online questionnaires and measured blood pressure using an automatic blood pressure monitor and body composition using a bioelectrical impedance analyzer at each visit, more details reported elsewhere [10,11].

**FIGURE 1:**
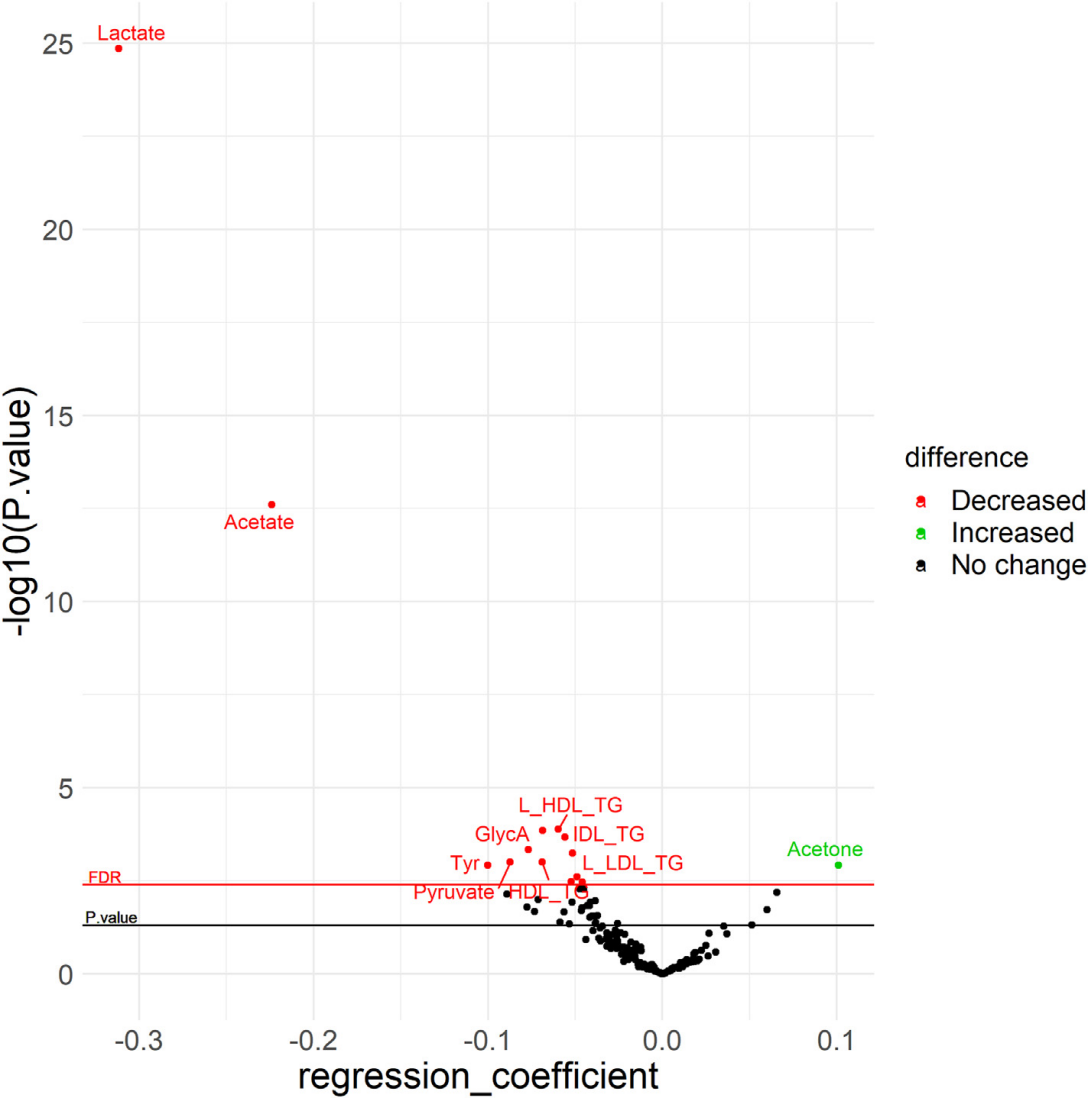
Significant changes in individual metabolites (normalized between 0 and 1) among LORANS participants after Ramadan fasting. GlycA, glycoprotein acetyls; HDL_TG, triglycerides in HDL; IDL_TG, triglycerides in intermediate-density lipoprotein; L_HDL_TG, triglycerides in large HDL; L_LDL_TG, triglycerides in large LDL; LDL_TG, triglycerides in LDL; LORANS, London Ramadan study; M_HDL_TG, triglycerides in medium HDL; Tyr, tyrosine; XS_VLDL_PL, phospholipids in very small VLDL; XS_VLDL_TG, triglycerides in very small VLDL.

All participants provided written informed consent before data collection. The study was approved by the Ethics Committee at Imperial College London (reference: 19IC5138, dated April 17, 2019).

#### Nightingale platform

Frozen aliquots of plasma (100 μL) were sent for targeted metabolic profiling analysis using high-throughput nuclear magnetic resonance (NMR) spectroscopy (Nightingale Health Ltd.), which quantifies 169 lipid and metabolites (https://nightingalehealth.com/biomarkers) from 17 substance classes: apolipoproteins (*n =* 2), amino acids (*n =* 10), cholesterol (*n =* 7), cholesteryl esters (*n =* 4), fatty acids (*n =* 9), fluid balance (*n =* 2), free cholesterol (*n =* 4), glycolysis-related metabolites (*n =* 5), inflammation (*n =* 1), ketone bodies (*n =* 4), lipoprotein particle concentrations (*n =* 4), lipoprotein particle sizes (*n =* 3), lipoprotein subclasses (*n =* 98), other lipids (*n =* 4), phospholipids (*n =* 4), total lipids (*n =* 4), and triglycerides (TGs, *n =* 4). The NMR-based metabolomics platform and the experimental procedure have been described in detail elsewhere [12].

#### UK Biobank

The UK Biobank is a large-scale prospective study with over 500,000 participants from across the United Kingdom. The study includes hospital inpatient data on diagnosis in the form of codes (ICD-9 and ICD-10) [13] with NMR metabolomic biomarkers data (Nightingale Health Ltd.) available on 117,981 UK Biobank participants [14].

#### Statistical analysis

##### Change of metabolite levels in LORANS

Metabolite levels were first normalized between 0 and 1 because normalizing data of different ranges is essential to compare results; this process transformed metabolites’ values to be between 0 and 1, reserving their unique variances. Next, we used a linear mixed-effects model for each metabolite to investigate the difference in its levels before and after Ramadan fasting. This model had age and sex as fixed effects and participant IDs, mosques, and day of the second measurement as random effects. To investigate whether the observed changes in metabolites were a result of changes in body composition, we constructed 2 additional models, further adjusting for: *1*) changes in waist circumference, free-fat mass, and BMI; *2*) changes in total body water and fat percentage. We applied Benjamini–Hochberg for false discovery rate (FDR) with a *q* value of 0.05 to correct for multiple testing [15].

##### The metabolic risk score for complex diseases

First, using data from the Nightingale platform in the UK Biobank, we used metabolite levels to construct metabolic risk scores for cardiometabolic disorders and incidence of a number of common cancers, namely coronary artery disease, diabetes, hypertension, renal failure, colorectal cancer, breast cancer, and lung cancer. These 7 diseases were selected because of their commonness and being the most likely to be affected by fasting. To this end, ICD-9 and ICD-10 codes from the hospital inpatient data available in the UK Biobank were used to define diseases within the subset of participants who have metabolomics data (*n =* 117,981) (Supplemental Table 2). For each disease of interest, the following steps were applied. Prevalent cases were excluded at baseline (2006). We examined the individual association of each metabolite (adjusted for age and sex) using a Cox proportional hazards model, adjusting *P* values for multiple testing by applying the FDR method. Metabolites associated with incident disease outcome at *P* < 0.05 were then included into a multivariate Cox proportional hazards model. We used the β coefficients from the models (1/metabolite) to produce a metabolic score per outcome per participant in LORANS. In cases of a high level of multicollinearity between variables in a model, we first applied a Cox Lasso regression to select variables that were then used in a multivariate Cox regression. Multicollinearity was assumed whenever the condition number (ratio of the largest singular value over the smallest singular value which is produced by the decomposition of the sample correlation matrix) was ≥30 [16]. Finally, from the multivariate Cox regression model output, we used the coefficients of the metabolites with *P* < 0.05 to generate a metabolic score for each participant in LORANS (Supplemental Figure 2). The following example clarifies this process further: to calculate the metabolic score of colorectal cancer for a participant in LORANS, we multiplied the regression coefficient (from the colorectal cancer multivariate Cox regression model) of glycoprotein acetyls (1 of the 2 metabolites contributing to this metabolic score) in the glycoprotein acetyls value assessed before Ramadan and added the result to that of free cholesterol in intermediate-density lipoprotein (IDL). This sum is considered the colorectal cancer metabolic score for that participant before Ramadan. Then, similarly, we calculate the score after Ramadan, from which the score before Ramadan was substracted to explore the difference (if any). The metabolic scores were rescaled so each participant would have a value between 0 and 100 using the “scales” package in R. The change in the estimated metabolic scores after Ramadan was examined using a mixed-effects model.

For lung cancer, we built a second metabolic score after further adjustment for smoking intensity in the initial univariate Cox regression model to adjust for the effect of smoking on metabolites.

Also, we built a metabolic score for smoking to investigate whether exposure to smoking changed during Ramadan. This was done by applying the same steps outlined above using a linear regression model.

We also assessed the correlation between metabolic scores and incidence rates of the 7 chronic diseases among the UK Biobank participants using a Cox proportional hazards regression model adjusted for age and sex. Moreover, we divided the metabolic scores into 4 groups (very low, low, high, and very high) and compared the frequency of incident cases across the 4 levels of the metabolic score.

We used “base,” “survival,” and “glmnet” packages in R (version 1.1-21) to perform statistical analyses. Also, we used complete-case analysis.

## Results

Table 1 shows the baseline characteristics of the study participants (*n* = 72). The mean age was 45.7 ± 16 y, and 35 of them were male (48.6%). Participants lost on average 1.7 kg [95% confidence interval (CI): 1.2, 1.9] and their body fat percentage reduced by 1.1% (95% CI: 0.4, 1.6) during Ramadan. Of the 169 metabolites measured using the Nightingale platform, after Ramadan fasting, there was a significant change in 14 metabolites surviving FDR correction (Table 2). Figure 1 summarizes the metabolites’ changes and their directions. These included 1 inflammation marker, 1 amino acid, 2 glycolysis-related metabolites, 2 ketone bodies, 2 TGs, and 6 lipoprotein subclasses. The most significant differences before/after Ramadan were observed for lactate (*β* = −0.31, *P* < 0.001), acetate (*β*= −0.22, *P* < 0.001), tyrosine (*β*= −0.10, *P* = 0.019) (all inverse), and acetone (*β* = 0.10, *P* = 0.019) (direct). These changes were independent of changes in body composition (Supplemental Table 3).

**TABLE 1:** Baseline characteristics of LORANS participants (*n =* 72)

Variable	Subgroups	Value
Age (y)	Total (mean ± SD)	45.7 (16)
	18–40 y (%)	21 (29.2%)
	40–60 y (%)	38 (52.8%)
	60–80 y (%)	11 (15.3%)
	>80 y (%)	2 (2.8%)
Sex (male %)	48.6%	
Ethnic background (%)	Somali	12 ( 16.7%)
	Pakistani	9 (12.5%)
	Indian	18 (25%)
	Bangladeshi	7 (9.7%)
	Arab	12 (16.7%)
	Other	9 (12.5%)
	Unknown	5 (6.9%)
Marital status (%)	Single	14 (19.4%)
	Married/living with a partner	50 (69.4%)
	Divorced/separated	3 (4.2%)
	Unknown	5 (7%)
With chronic diseases (%)	Diabetes	8 (11.1%)
	Hypertension	15 (20.8%)
	Cardiovascular diseases	4 (5**.**6%)
Education (%)	No formal qualification	9 (12.5%)
	Secondary school or equivalent	16 (22.2%)
	Higher education: college/HNC/HND	15 (20.8%)
	Vocational qualification	1 (1.4%)
	Bachelor’s degree	18 (25%)
	Postgraduate degree	8 (11.1%)
	Unknown	5 (6.9%)
Smoking (%)	Never	55 (76**.**4%)
	Stopped	10 (13.9%)
	Occasionally	4 (5**.**5%)
	Yes, most or all days	3 (4.2%)

Abbreviation: HNC, Higher National Certificate; HND, Higher National Diploma; LORANS, London Ramadan study.

**TABLE 2:** The 14 metabolites changed significantly (*P* < 0.05) after Ramadan fasting (normalized between 0 and 1)

Metabolic biomarker	Class	Change after Ramadan (95% CI)^1^	*P* value	FDR adjustment
Lactate	Glycolysis-related metabolites	−0.31 (−0.36, −0.26)	<0.001	<0.001
Acetate	Ketone bodies	−0.22 (−0.27, −0.18)	<0.001	<0.001
Glycoprotein acetyls	Inflammation marker	−0.07 (−0.1, −0.04)	<0.001	0.006
Triglycerides in large HDL	Lipoprotein subclasses	−0.06 (−0.09, −0.03)	<0.001	0.006
Triglycerides in IDL	Lipoprotein subclasses	−0.06 (−0.11, −0.05)	<0.001	0.007
Triglycerides in medium HDL	Lipoprotein subclasses	−0.08 (−0.12, −0.04)	<0.001	0.013
Triglycerides in large LDL	Lipoprotein subclasses	−0.05 (−0.08, −0.02)	<0.001	0.014
Triglycerides in HDL	Triglycerides	−0.07 (−0.11, −0.03)	0.001	0.019
Tyrosine	Amino acids	−0.1 (−0.16, −0.04)	0.001	0.019
Pyruvate	Glycolysis-related metabolites	−0.09 (−0.14, −0.04)	0.001	0.019
Acetone	Ketone bodies	0.10 (0.04, 0.16)	0.001	0.019
Triglycerides in LDL	Triglycerides	−0.05 (−0.08, −0.02)	0.003	0.035
Phospholipids in very small VLDL	Lipoprotein subclasses	−0.05 (−0.08, −0.02)	0.003	0.041
Triglycerides in very small VLDL	Lipoprotein subclasses	−0.05 (−0.09, −0.02)	0.003	0.041

Abbreviations: CI, confidence interval; FDR, false discovery rate; IDL, intermediate-density lipoprotein.

1 Adjusted for age, sex, mosques, and day of the second measurement.

**FIGURE 2:**
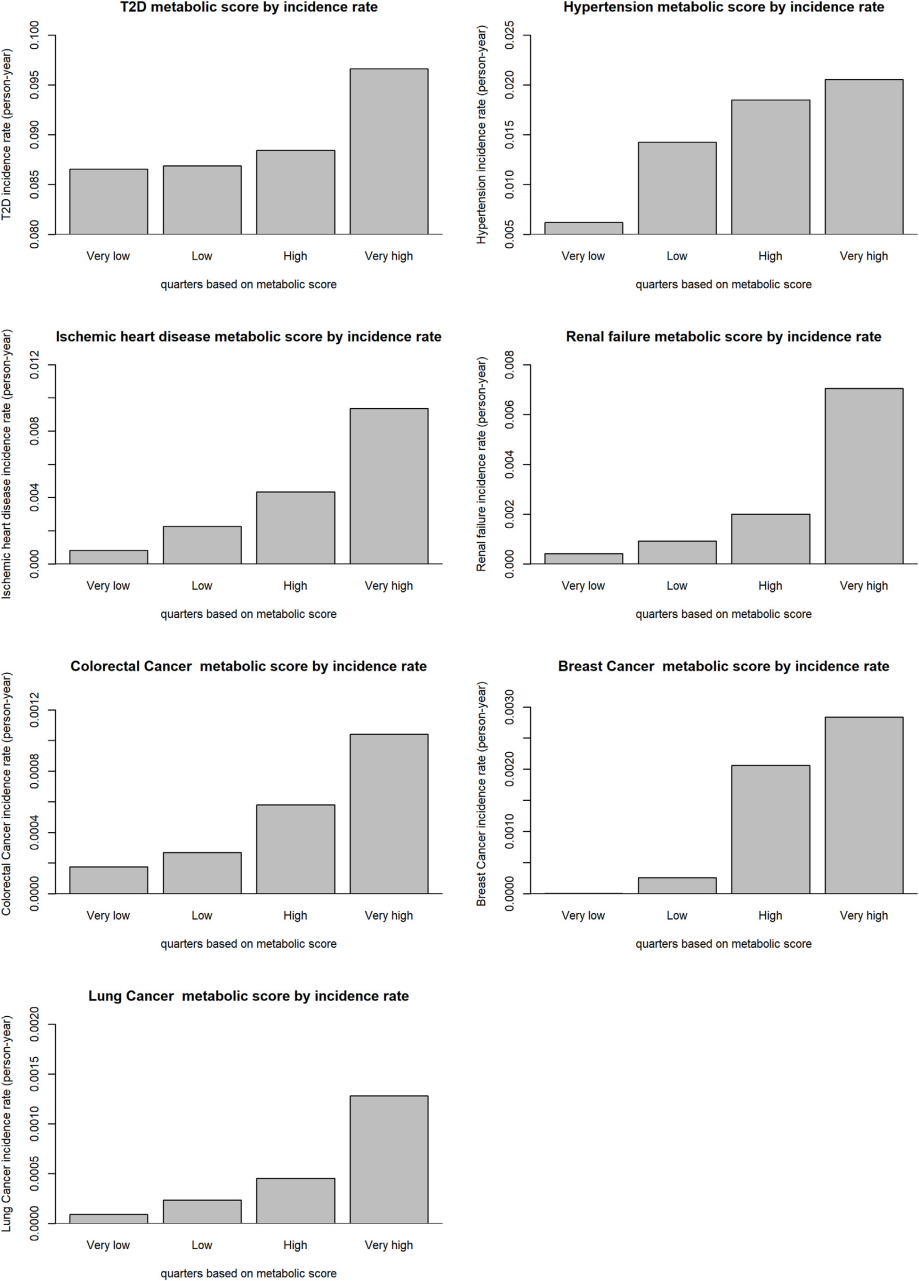
Correlation between metabolic scores and incidence rates of chronic diseases in the UK Biobank.

**FIGURE 3:**
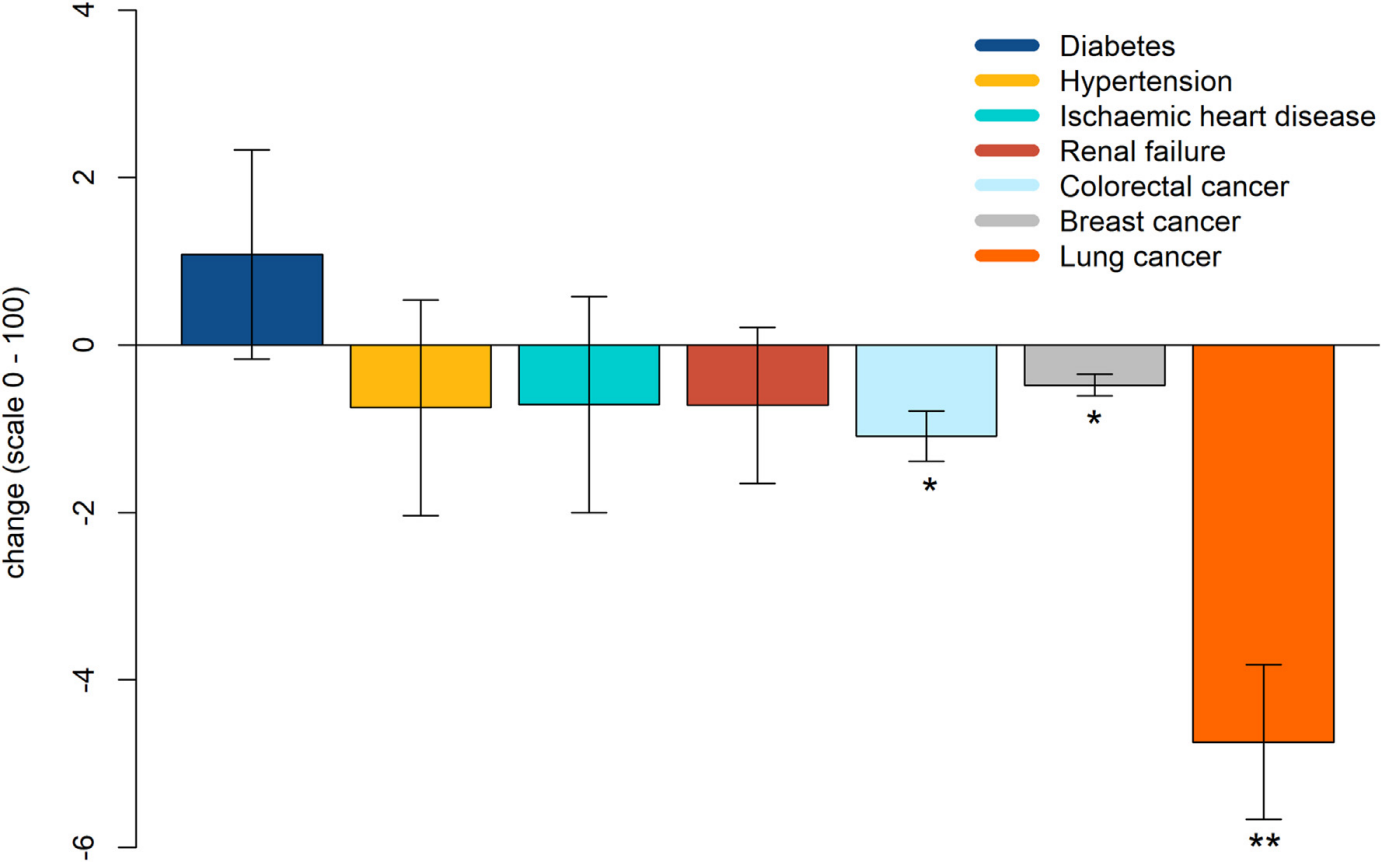
Changes in metabolic scores among LORANS participants after Ramadan fasting. LORANS, London Ramadan study.

Baseline characteristics of 117,981 participants whose metabolic biomarkers were measured using NMR spectroscopy in the UK Biobank are shown in Table 3. Using the UK Biobank data, we generated 7 metabolic scores: for diabetes (using 46 metabolites), hypertension (25 metabolites), coronary artery disease (16 metabolites), renal failure (12 metabolites), colorectal cancer (2 metabolites), breast cancer (1 metabolite), and lung cancer (9 metabolites) (Table 4). The increase in metabolic scores was associated with an increase in incidence rates of the 7 cases among the the UK Biobank participants as shown in Supplemental Table 4. This association was clearly displayed in Figure 2 where the metabolic scores were divided into 4 quarters and an incremental increase in a number of cases associated with higher metabolic scores. As shown in Figure 3, the metabolic score for lung cancer decreased after Ramadan (−4.74, −9.6%, 95% CI: −6.56, −2.91) and (−1.09, −2.4%, 95% CI: −1.69, −0.50) for colorectal cancer (Supplemental Table 5), with glycoprotein acetyls being the main driver of these changes (significance was lost when the models were performed excluding this inflammation biomarker) (Supplemental Table 6). Breast cancer metabolic score decreased after Ramadan; however, it consisted of only 1 metabolite (−0.48, −1.1%, 95% CI: −0.81, −0.1). We reported nonsignificant changes in the metabolic scores for diabetes (1.08, 95% CI: −2.40, 4.58), hypertension (−0.75, 95% CI: −3.35, 1.86), coronary artery disease (−0.71, 95% CI: −3.33, 1.91), and renal failure (−0.72, 95% CI: −3.34, 1.89). Supplemental Figure 3 shows the changes in the metabolic scores for each LORANS participant.

**TABLE 3:** Baseline characteristics of the UK Biobank participants with metabolomics data (*n =* 117,981)

Variable	Subgroups	Value
	Total (mean ± SD)	56.5 ± 8.1
Age (y)	39–50 y (%)	27,676 (23.5%)
	50–60 y (%)	39,269 (33.3%)
	60–71 y (%)	51,036 (43.2%)
Sex (male%)	45.8%	
BMI (kg/m^2^)	27.4 ± 4.8	
Systolic blood pressure (mm Hg)	137.6 ± 18.5	
Diastolic blood pressure (mm Hg)	82.1 ± 10.1	
Creatinine (μmol/L)	72.3 ± 18.7	
eGFR	90.7 ± 13.4	
Prevalence rate at baseline	T2D	2683 (2.3%)
	Hypertension	9486 (8%)
	coronary artery disease	4844 (4.1%)
	CKD (eGFR <60 mL/min/1.73 m^2^)	448 (0.4%)
	Colorectal cancer	321 (0.3%)
	Breast cancer	1772 (1.5%)
	Lung cancer	84 (0.07%)

Abbreviations: BMI, body mass index; CKD, chronic kidney disease; eGFR, estimated glomerular filtration rate; mm Hg, Millimetre of mercury; T2D, type 2 diabetes; μmol/L, micromoles per litre.

**TABLE 4:** Number of metabolites correlated with/contributing to the metabolic scores

Disease	Total number of participants free of disease in UK Biobank (of 117,981)	Total number of incident cases (of 117,981)	Number of metabolites highly correlated with the disease using a univariate model	Number of metabolites contributing to metabolic score
Diabetes	110,598	4700	157	46
Hypertension	91,456	17,039	154	25
coronary artery disease	107,796	5341	150	16
Renal failure	114,062	3471	158	12
Colorectal cancer	116,943	717	18	2
Breast cancer	114,460	1749	3	1
Lung cancer	117,184	713	88	9

Selecting the metabolites associated with lung cancer in the UK Biobank adjusted for smoking intensity showed that the effect on lung cancer score was independent of smoking (−5.69%; −2.95; and −4.74, −1.18) (Supplemental Table 7). Also, the smoking metabolic score we created did not change significantly, meaning there was no change in smoking during Ramadan (Supplemental Table 8).

## Discussion

In this study, we investigated the metabolic changes after fasting in the month of Ramadan. We found changes in serum levels of 14 metabolites. Using 7 metabolic risk scores that were built using data from the UK Biobank, we showed that the metabolic risk scores were lower for lung cancer and, to a lesser extent, for colorectal cancer and breast cancer after 1 mo of fasting in Ramadan. However, this study does not provide evidence for an association between Ramadan fasting and a lower risk of these diseases. The findings merely showed changes in the metabolic profile, similar to that of individuals at lower risk of the diseases.

Mathew et al. [8] measured 202 metabolite levels by applying extended targeted metabolomics assays to dried blood spots for 11 men during Ramadan. Twenty metabolites, mostly phosphatidylcholines, were significantly changed from the first to the fourth week of Ramadan. However, phosphatidylcholines were measured in LORANS as one broad class and did not change after Ramadan fasting. Only 2 (lactate and tyrosine) of the 14 metabolites that changed significantly in LORANS were measured in Mathew et al.’s [8] study, and none was reported to be changed. Notably, the 2 studies (LORANS and Mathew) collected the first and second blood samples at different times and used different metabolomics profiling methods, which might have contributed to the differences observed in the metabolite changes.

A systematic review and meta-analysis of data on healthy individuals showed that Ramadan fasting is associated with reduced HDL but no changes in TGs, total cholesterol, or LDL [17]. In contrast, we do not report reductions in HDL, which may partly be explained by LORANS participants being recruited from a community-based sample including healthy and unhealthy individuals, whereas the review only involved studies on apparently healthy individuals.

### Implication on public health and potential consequences

Ketogenesis has been mostly characterized in the context of ketogenic diets, showing positive outcomes for the microbiome, lowering hemoglobin A1c (HbA1c) and the need to reduce insulin in diabetics, helping weight loss, reducing visceral adiposity, positively regulating lipoproteins, lowering TGs, and reducing inflammation [18,19].

In addition, the observed metabolic changes have potential consequences. The increase in acetone and decrease of acetate, together with decreases in both lactate and pyruvate as well as decreasing lipoprotein TGs point to an energy pathway shift in the liver after Ramadan fasting, from glucose to fatty acid metabolism. This can be seen at 3 levels:

1) In ketogenesis, the limited availability of oxaloacetate (because of its consumption in parallel with gluconeogenesis) leads to the production of ketone bodies from acetyl coenzyme A resulting from the breakdown of fatty acids.
2) Pyruvate is the product of glycolysis and a precursor to the Krebs cycle for energy production, whereas lactate is its by-product. The observed reduction in both pyruvate and lactate can thus be explained by the shift from glycolysis to ketogenesis.
3) There is a reduction of fatty acid-rich TGs in multiple lipoprotein fractions, which reflects lipolysis and breakdown of fatty acids during ketogenesis.

### Fasting and cancer

In this study, we found that a metabolic risk score associated with the risk of lung cancer and colorectal cancer is reduced after Ramadan fasting. A number of experimental studies on animal models have provided evidence for the preventive effect of fasting on developing cancer [[20], [21], [22]].

Previous studies have reported associations between some of the metabolites that we have used in the metabolic risk score and lung cancer, such as alanine and β-hydroxybutyrate in Zhang et al.’s [23] study and citrate, alanine, and lactate in Rocha et al.’s [24] study.

### Glycoprotein acetyls

Glycoprotein acetyls was 1 of 9 metabolite measures contributing to the metabolic score of lung cancer and 1 of 2 variables that constituted the colorectal cancer metabolic score. Glycoprotein acetyls signal is mainly influenced by concentrations of 5 circulating acute-phase inflammatory glycoproteins [25]. Previous studies have identified glycoprotein acetyls as a predictive marker for diabetes [26], cardiovascular diseases [27], all-cause mortality [28], total cancers [29], colorectal cancer [30], and cancer deaths [28]. In our study, glycoprotein acetyls contributed to the metabolic scores of diabetes, hypertension, coronary artery disease, renal failure, colorectal cancer, and lung cancer. In addition, glycoprotein acetyls appeared to be the main driver of reductions in colorectal cancer and lung cancer metabolic scores after Ramadan fasting (Supplemental Table 6).

### Impact of smoking on metabolic profiles

Because smoking is a major risk factor for lung cancer and is known to affect the metabolic profile [31], we conducted a number of sensitivity analyses to minimize any bias because of confounding by smoking. Unfortunately, the small number of smokers in LORANS did not allow adjusting for smoking. The small number of smokers might be because of misclassification in the collected data on smoking, because it is prohibited in Islam and is not socially accepted. Also, some Muslims find Ramadan an excellent opportunity to quit smoking; thus, some former smokers may have stopped smoking a few days before taking part in the study. However, the effect of fasting on lung cancer metabolic risk remained significant after constructing another metabolic score adjusted for smoking in the UK Biobank, and the metabolic score for smoking did not change after Ramadan fasting (Supplemental Tables 7 and 8), suggesting that smoking is not likely to be an important confounder in the fasting/metabolite relationships.

### Strength and weaknesses

This study has several strengths. First, LORANS has recruited a sample from the general population with different ethnic backgrounds. Studies that have been done on each of these ethnic groups in their home countries recruited participants from different socioeconomic statuses that might affect the lifestyle changes during Ramadan and cause discrepancies across studies. In LORANS, we have a sample with similar socioeconomic status because the participants were recruited from similar neighborhoods of the same city. This makes the results of the study more generalizable compared with previous studies. Second, we used the Nightingale NMR platform that covers a range of metabolites [8]. We also used the Nightingale platform to estimate metabolic risk scores in the UK Biobank study with ∼120,000 participants, enabling us to create robust metabolic scores and extrapolate the metabolic changes to the risk of a range of clinical outcomes.

This study also has some limitations. First, blood samples from 68 individuals were not available for follow-up. Another limitation is that we did not apply cross-validation to the Cox Lasso regression model.

In conclusion, Ramadan fasting is associated with short-term favorable changes in the metabolic profile concerning the risk of some chronic diseases. These findings should be further investigated in future, larger studies of longer follow-up with clinical outcomes.

## Acknowledgments

PE is the Director of the MRC Centre for Environment and Health and acknowledges support from the Medical Research Council (MR/L01341X/1 and MR/S019669/1). PE also acknowledges support from the National Institute for Health Research NIHR Imperial Biomedical Research Centre, Imperial College London; and the British Heart Foundation Imperial College Centre for Research Excellence. PE, AD, and RCP are associated with the UK DRI at the UK Dementia Research Institute, Imperial College London, which receives funding from the Medical Research Council, Alzheimer’s Society and Alzheimer’s Research UK.

## Author contributions

The authors’ responsibilities were as follows – RA-J, AD, KKT: designed the research; RA-J: conducted the research; RA-J, AD: provided essential materials; RA-J: analyzed the data; RA-J: wrote the manuscript; AD, KKT, RCP, PE: critically reviewed the manuscript and provided feedback; RA-J: wrote the article; RA-J: had primary responsibility for final content; and all authors: read and approved the final manuscript.

## Conflicts of interest

The authors report no conflicts of interest.

## Funding

This work was partially funded by the Saudi Arabia Cultural Bureau in London. The funder had no role in either the study design, data collection, data analysis, data interpretation, or the decision to publish the study.

## Data availability

Data described in the manuscript and analytic code will be made available upon request pending [application and approval].

### Ethical approval

The study is ethically approved by the Imperial College Research Ethics Committee (reference: 19IC5138, dated: April 17, 2019).

### Patient consent

All participants gave informed consent before taking part in LORANS.
