# Supplementary material for: Metabolomics of Ramadan fasting and associated risk of chronic diseases
Source: Am J Clin Nutr. 2024 Feb 1;119(4):1007–14. doi: 10.1016/j.ajcnut.2024.01.019 (PMC11007737; doi:10.1016/j.ajcnut.2024.01.019)
Supplement: Multimedia component 1 [file mmc1.docx]

**Supplemental File for publication**

**Title of manuscript: Metabolomics of Ramadan fasting and associated risk of chronic diseases**

First Author: Rami Al-Jafar (<https://orcid.org/0000-0002-9393-7585>)


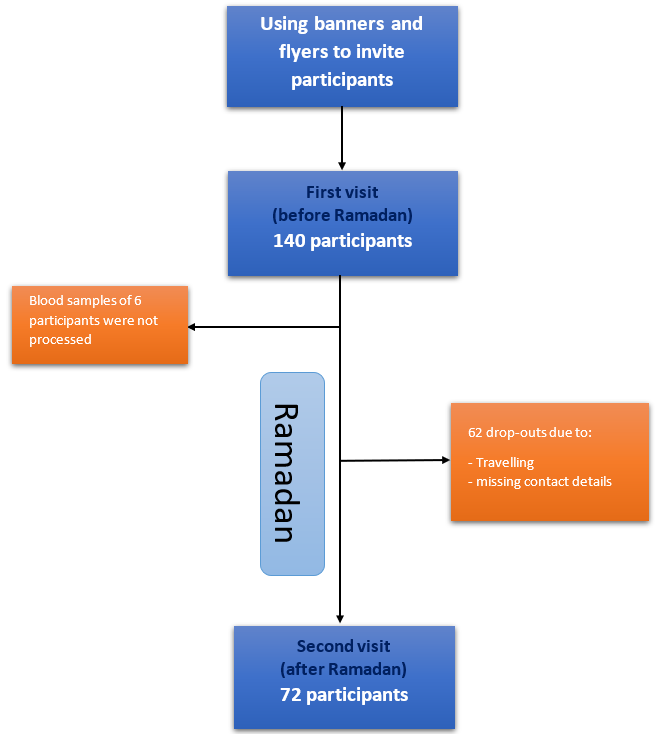


**Supplemental Figure 1:** flowchart of participants' attendance to provide blood samples at the first and the second visit in LORANS.


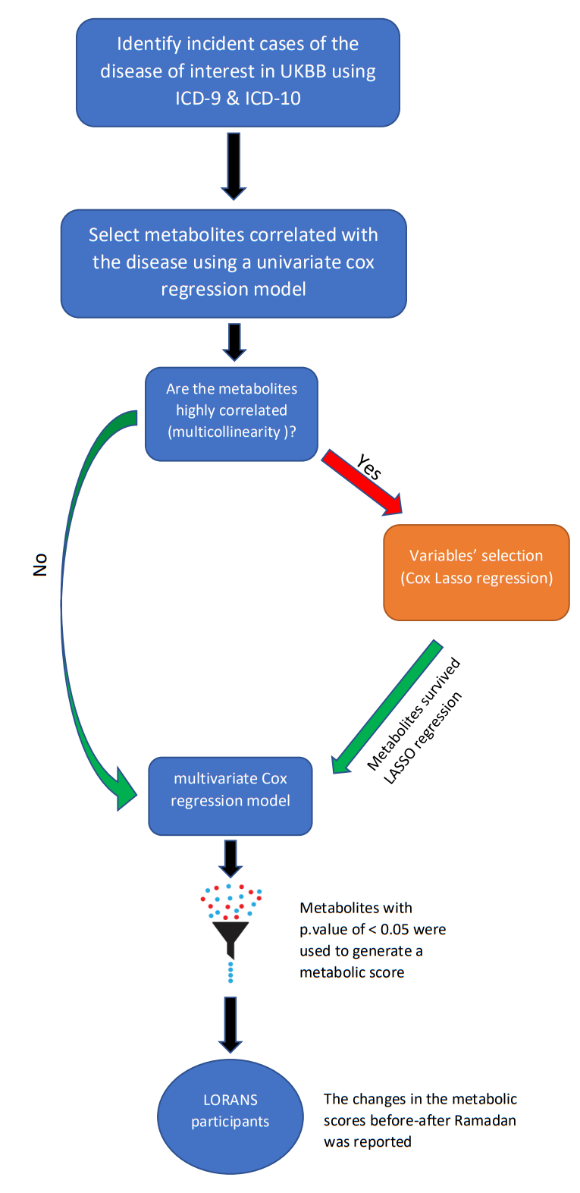


**Supplemental Figure 2:** The process of generating risk scores for diseases.


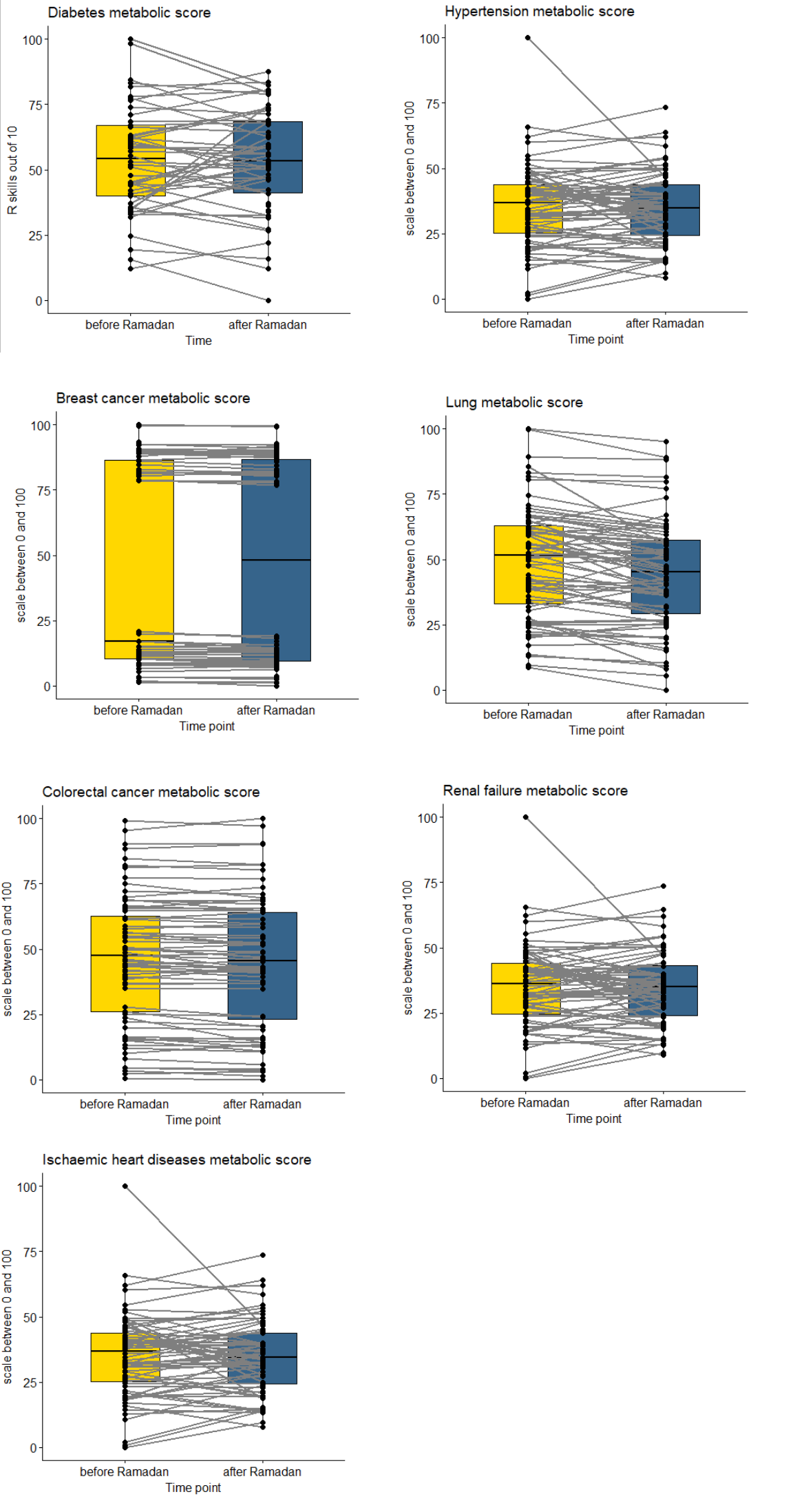


**Supplemental Figure 3:** changes in metabolic scores after Ramadan fasting per participant.

**Supplemental Table 1:** Comparison of the study sample and individuals who dropped out.

| **Variable** | **Sub-groups** | **Study participants (n=72)** | **Individuals who didn’t give a second blood sample (n=68)** | **p-value** |
| --- | --- | --- | --- | --- |
| **Age**  **(mean ± SD)** | Total | 45.7 ± 15.6 | 40 ± 22 ^꙰^ | 0.005 |
|  | 18 – 40 years (%) | 30.6% | 51.5% |  |
|  | 40 – 60 years (%) | 52.7% | 38.2% |  |
|  | 60 – 80 years (%) | 15.3% | 10.3% |  |
|  | > 80 years (%) | 1.4% | 0% |  |
| **Gender (male %)** | | 51.4% | 52.9 % | 0.74 |
| **Ethnic background**  **(%)** | Pakistani | 13.4% | 24.5% | 0.14 |
|  | Indian | 26.9% | 44% |  |
|  | Bangladeshi | 10.4% | 5.7% |  |
|  | Somali | 17.9% | 18.9% |  |
|  | Arab | 17.9% | 3.8% |  |
|  | Other | 13.4% | 13.2% |  |
| **Marital status**  **(%)** | Single | 20.9% | 32.1% | 0.311 |
|  | Married/living with a partner | 74.6 % | 66% |  |
|  | Divorced/separated | 4.5 % | 1.9% |  |
| **With Chronic diseases**  **(%)** | Diabetes | 11.9% | 7.4% | 0.60 |
|  | Hypertension | 22.4% | 8.8% | 0.80 |
|  | Cardiovascular diseases | 6% | 3% | 0.43 |
| **Education**  **(%)** | No formal qualification | 13.4% | 9.4% | 0.28 |
|  | Secondary school or equivalent | 23.9% | 18.9% |  |
|  | Higher education: College/HNC/HND | 22.4% | 13.2% |  |
|  | Vocational qualification | 1.5% | 7.5% |  |
|  | Bachelor’s degree | 26.9% | 3.2% |  |
|  | Postgraduate degree | 11.9% | 20.8% |  |
| **Smoking**  **(%)** | Never | 76.4% | 70.6% | 0.08 |
|  | Stopped | 15.3% | 2.9% |  |
|  | Occasionally | 4.2% | 4.4% |  |
|  | Yes, most or all days | 4.2% | 1.5% |  |

**Supplemental Table 2:** ICD-9 and ICD-10 codes used to define diseases within the subset of UK Biobank participants.

| **Disease** | **ICD-9** | **ICD-10** |
| --- | --- | --- |
| **Diabetes** | Codes start with “250” | E10, E11, E12, E13, E14 |
| **Hypertension** | 4019 | I10 |
| **coronary heart disease** | 4109, 4119, 4129, 4139, 414x | I20, I21, I22, I23, I24, I25 |
| **Renal failure** | 5845, 5849, 5859, 5869 | N17.x, N18.x, N19 |
| **Colorectal cancer** | 1530, 1532, 1533, 1534, 1536, 1537, 1539 | C18.x |
| **Breast cancer** | 174x | C50.x ; D05.x |
| **Lung cancer** | 1623; 1629 | C34.x |

x= a number between 0 and 9.

**Supplemental Table 3:** Changes in metabolites with and without adjustments for body composition parameters using mixed-effects model.

| **metabolite** | **Base model: Beta (CI)** | **Base model FDR adjusted p.value** | **model i: Beta (CI)** | **model i: FDR adjusted p.value** | **model ii: Beta (CI)** | **model ii: FDR adjusted p.value** |
| --- | --- | --- | --- | --- | --- | --- |
| **Lactate** | -0.31 (-0.36 to -0.26) | <0.001 | -0.31 (-0.36 to NA) | <0.001 | -0.31 (-0.36 to 0.89) | <0.001 |
| **Acetate** | -0.22 (-0.27 to -0.18) | <0.001 | -0.23 (-0.28 to NA) | <0.001 | -0.22 (-0.27 to 0.6) | <0.001 |
| **Glycoprotein acetyls** | -0.07 (-0.1 to -0.04) | 0.006 | -0.06 (-0.1 to NA) | 0.048 | -0.06 (-0.09 to 0.52) | 0.036 |
| **Triglycerides in large HDL** | -0.06 (-0.09 to -0.03) | 0.006 | -0.06 (-0.1 to NA) | 0.018 | -0.07 (-0.1 to 0.8) | 0.002 |
| **Triglycerides in IDL** | -0.06 (-0.08 to -0.03) | 0.007 | -0.05 (-0.09 to NA) | 0.048 | -0.06 (-0.09 to 0.65) | 0.012 |
| **Triglycerides in medium HDL** | -0.08 (-0.12 to -0.04) | 0.013 | -0.07 (-0.12 to NA) | 0.051 | -0.08 (-0.13 to 0.84) | 0.012 |
| **Triglycerides in large LDL** | -0.05 (-0.08 to -0.02) | 0.014 | -0.05 (-0.08 to NA) | 0.076 | -0.05 (-0.08 to 0.61) | 0.030 |
| **Acetone** | 0.1 (0.04 to 0.16) | 0.019 | 0.1 (0.04 to NA) | 0.051 | 0.1 (0.04 to 0.48) | 0.030 |
| **Triglycerides in HDL** | -0.07 (-0.11 to -0.03) | 0.019 | -0.07 (-0.11 to NA) | 0.066 | -0.08 (-0.12 to 0.82) | 0.020 |
| **Pyruvate** | -0.09 (-0.14 to -0.04) | 0.019 | -0.09 (-0.15 to NA) | 0.043 | -0.08 (-0.14 to 0.43) | 0.045 |
| **Tyrosine** | -0.1 (-0.16 to -0.04) | 0.019 | -0.09 (-0.15 to NA) | 0.064 | -0.08 (-0.14 to 0.44) | 0.082 |
| **Triglycerides in LDL** | -0.05 (-0.08 to -0.02) | 0.035 | -0.04 (-0.08 to NA) | 0.142 | -0.05 (-0.09 to 0.63) | 0.057 |
| **Phospholipids in very small VLDL** | -0.05 (-0.08 to -0.02) | 0.041 | -0.04 (-0.07 to NA) | 0.190 | -0.05 (-0.08 to 0.75) | 0.057 |
| **Triglycerides in very small VLDL** | -0.05 (-0.09 to -0.02) | 0.041 | -0.05 (-0.09 to NA) | 0.134 | -0.06 (-0.09 to 0.68) | 0.050 |
| **Total lipids in very small VLDL** | -0.05 (-0.08 to -0.01) | 0.056 | -0.04 (-0.07 to NA) | 0.237 | -0.05 (-0.08 to 0.78) | 0.078 |
| **Concentration of very small VLDL particles** | -0.05 (-0.08 to -0.02) | 0.056 | -0.04 (-0.08 to NA) | 0.237 | -0.05 (-0.08 to 0.79) | 0.082 |
| **Glutamine** | 0.07 (0.02 to 0.11) | 0.064 | 0.06 (0.01 to NA) | 0.170 | 0.07 (0.02 to 0.42) | 0.070 |
| **Acetoacetate** | -0.09 (-0.15 to -0.03) | 0.068 | -0.08 (-0.14 to NA) | 0.190 | -0.08 (-0.15 to 0.43) | 0.118 |
| **Alanine** | -0.07 (-0.12 to -0.02) | 0.089 | -0.08 (-0.14 to NA) | 0.079 | -0.07 (-0.13 to 0.5) | 0.093 |
| **Phospholipids in medium HDL** | -0.05 (-0.09 to -0.01) | 0.093 | -0.05 (-0.09 to NA) | 0.258 | -0.05 (-0.09 to 0.84) | 0.164 |
| **Triglycerides in very large HDL** | -0.04 (-0.07 to -0.01) | 0.093 | -0.04 (-0.07 to NA) | 0.142 | -0.05 (-0.08 to 0.8) | 0.045 |
| **Free cholesterol in very small VLDL** | -0.04 (-0.07 to -0.01) | 0.093 | -0.03 (-0.07 to NA) | 0.307 | -0.04 (-0.08 to 0.76) | 0.135 |
| **Phospholipids in HDL** | -0.04 (-0.08 to -0.01) | 0.106 | -0.04 (-0.08 to NA) | 0.237 | -0.04 (-0.08 to 0.9) | 0.137 |
| **Triglycerides in medium LDL** | -0.04 (-0.07 to -0.01) | 0.106 | -0.04 (-0.07 to NA) | 0.280 | -0.04 (-0.08 to 0.6) | 0.164 |
| **Leucine** | -0.08 (-0.14 to -0.02) | 0.109 | -0.08 (-0.14 to NA) | 0.170 | -0.06 (-0.13 to 0.44) | 0.217 |
| **Triglycerides in small HDL** | -0.05 (-0.08 to -0.01) | 0.111 | -0.04 (-0.08 to NA) | 0.280 | -0.05 (-0.09 to 0.62) | 0.137 |
| **Glucose** | 0.06 (0.01 to 0.11) | 0.120 | 0.06 (0.01 to NA) | 0.152 | 0.07 (0.02 to 0.31) | 0.089 |
| **Total lipids in medium HDL** | -0.05 (-0.08 to -0.01) | 0.121 | -0.04 (-0.08 to NA) | 0.280 | -0.04 (-0.08 to 0.79) | 0.201 |
| **Phenylalanine** | -0.07 (-0.13 to -0.01) | 0.123 | -0.08 (-0.14 to NA) | 0.142 | -0.07 (-0.13 to 0.57) | 0.184 |
| **Albumin** | -0.06 (-0.1 to -0.01) | 0.125 | -0.06 (-0.11 to NA) | 0.142 | -0.05 (-0.1 to 0.94) | 0.184 |
| **Apolipoprotein A1** | -0.04 (-0.08 to -0.01) | 0.144 | -0.03 (-0.07 to NA) | 0.324 | -0.04 (-0.07 to 0.75) | 0.251 |
| **Total lipids in HDL** | -0.04 (-0.07 to 0) | 0.144 | -0.03 (-0.07 to NA) | 0.280 | -0.04 (-0.07 to 0.87) | 0.184 |
| **Cholesterol in very small VLDL** | -0.04 (-0.07 to -0.01) | 0.144 | -0.03 (-0.07 to NA) | 0.418 | -0.04 (-0.08 to 0.85) | 0.201 |
| **Concentration of medium HDL particles** | -0.04 (-0.08 to 0) | 0.150 | -0.04 (-0.08 to NA) | 0.324 | -0.04 (-0.08 to 0.76) | 0.259 |
| **Total concentration of branched-chain amino acids (leucine + isoleucine + valine)** | -0.06 (-0.11 to 0) | 0.198 | -0.06 (-0.11 to NA) | 0.280 | -0.05 (-0.1 to 0.41) | 0.367 |
| **Cholesteryl esters in very small VLDL** | -0.04 (-0.07 to 0) | 0.198 | -0.03 (-0.07 to NA) | 0.482 | -0.04 (-0.08 to 0.9) | 0.259 |
| **Average diameter for HDL particles** | -0.03 (-0.05 to 0) | 0.201 | -0.03 (-0.06 to NA) | 0.190 | -0.04 (-0.06 to 1.12) | 0.078 |
| **Phosphoglycerides** | -0.04 (-0.08 to 0) | 0.201 | -0.03 (-0.07 to NA) | 0.482 | -0.03 (-0.07 to 0.82) | 0.378 |
| **Valine** | -0.05 (-0.1 to 0) | 0.201 | -0.05 (-0.1 to NA) | 0.370 | -0.04 (-0.09 to 0.44) | 0.419 |
| **Free cholesterol in small LDL** | 0.05 (0 to 0.1) | 0.208 | 0.07 (0.02 to NA) | 0.131 | 0.07 (0.02 to 0.62) | 0.080 |
| **Triglycerides in large VLDL** | 0.04 (0 to 0.07) | 0.215 | 0.04 (0 to NA) | 0.280 | 0.03 (0 to 0.42) | 0.275 |
| **Cholesteryl esters in small VLDL** | -0.03 (-0.07 to 0) | 0.215 | -0.02 (-0.06 to NA) | 0.518 | -0.03 (-0.07 to 0.66) | 0.419 |
| **Total cholines** | -0.04 (-0.07 to 0) | 0.233 | -0.02 (-0.06 to NA) | 0.518 | -0.03 (-0.07 to 0.8) | 0.419 |
| **Phospholipids in large HDL** | -0.03 (-0.06 to 0) | 0.263 | -0.03 (-0.06 to NA) | 0.280 | -0.04 (-0.07 to 1.11) | 0.176 |
| **Phospholipids in small HDL** | -0.04 (-0.08 to 0) | 0.264 | -0.02 (-0.07 to NA) | 0.608 | -0.03 (-0.07 to 0.6) | 0.459 |
| **Free cholesterol in HDL** | -0.03 (-0.06 to 0) | 0.273 | -0.03 (-0.06 to NA) | 0.400 | -0.03 (-0.06 to 0.92) | 0.276 |
| **Total lipids in large HDL** | -0.02 (-0.05 to 0) | 0.281 | -0.03 (-0.06 to NA) | 0.292 | -0.03 (-0.06 to 1.09) | 0.184 |
| **Free cholesterol in medium HDL** | -0.03 (-0.07 to 0) | 0.281 | -0.03 (-0.07 to NA) | 0.452 | -0.03 (-0.07 to 0.78) | 0.410 |
| **Average diameter for VLDL particles** | 0.03 (0 to 0.06) | 0.281 | 0.03 (0 to NA) | 0.280 | 0.03 (0 to 0.58) | 0.305 |
| **Phospholipids in small LDL** | 0.04 (0 to 0.08) | 0.286 | 0.05 (0.01 to NA) | 0.190 | 0.05 (0.01 to 0.63) | 0.151 |
| **Concentration of large HDL particles** | -0.02 (-0.05 to 0) | 0.290 | -0.03 (-0.05 to NA) | 0.307 | -0.03 (-0.06 to 1.07) | 0.184 |
| **Monounsaturated fatty acids** | -0.03 (-0.06 to 0) | 0.294 | -0.02 (-0.06 to NA) | 0.608 | -0.02 (-0.06 to 0.54) | 0.425 |
| **Triglycerides in small LDL** | -0.03 (-0.06 to 0) | 0.321 | -0.02 (-0.06 to NA) | 0.482 | -0.03 (-0.06 to 0.55) | 0.340 |
| **Concentration of IDL particles** | -0.04 (-0.08 to 0.01) | 0.340 | -0.01 (-0.06 to NA) | 0.750 | -0.03 (-0.07 to 0.82) | 0.530 |
| **Cholesterol in medium HDL** | -0.03 (-0.07 to 0.01) | 0.340 | -0.03 (-0.07 to NA) | 0.482 | -0.03 (-0.07 to 0.76) | 0.476 |
| **Isoleucine** | -0.04 (-0.1 to 0.01) | 0.352 | -0.05 (-0.1 to NA) | 0.390 | -0.03 (-0.09 to 0.37) | 0.506 |
| **Cholesteryl esters in medium HDL** | -0.03 (-0.07 to 0.01) | 0.352 | -0.03 (-0.07 to NA) | 0.486 | -0.03 (-0.07 to 0.77) | 0.506 |
| **Total phospholipids in lipoprotein particles** | -0.03 (-0.07 to 0.01) | 0.352 | -0.02 (-0.06 to NA) | 0.662 | -0.02 (-0.07 to 0.9) | 0.506 |
| **Polyunsaturated fatty acids** | -0.03 (-0.07 to 0.01) | 0.362 | -0.02 (-0.06 to NA) | 0.651 | -0.02 (-0.06 to 0.66) | 0.615 |
| **Total fatty acids** | -0.03 (-0.06 to 0.01) | 0.362 | -0.01 (-0.05 to NA) | 0.665 | -0.02 (-0.06 to 0.65) | 0.516 |
| **Degree of unsaturation** | -0.04 (-0.08 to 0.01) | 0.362 | -0.03 (-0.08 to NA) | 0.482 | -0.02 (-0.07 to 0.57) | 0.620 |
| **Free cholesterol in large HDL** | -0.02 (-0.04 to 0.01) | 0.372 | -0.02 (-0.05 to NA) | 0.400 | -0.03 (-0.05 to 1.05) | 0.245 |
| **Linoleic acid** | -0.03 (-0.06 to 0.01) | 0.372 | -0.02 (-0.06 to NA) | 0.625 | -0.02 (-0.06 to 0.65) | 0.561 |
| **Omega-6 fatty acids** | -0.03 (-0.07 to 0.01) | 0.372 | -0.02 (-0.06 to NA) | 0.662 | -0.02 (-0.06 to 0.66) | 0.620 |
| **Phosphatidylcholines** | -0.03 (-0.06 to 0.01) | 0.392 | -0.02 (-0.05 to NA) | 0.655 | -0.02 (-0.06 to 0.86) | 0.506 |
| **Cholesterol in small VLDL** | -0.03 (-0.06 to 0.01) | 0.406 | -0.01 (-0.05 to NA) | 0.708 | -0.02 (-0.06 to 0.68) | 0.605 |
| **Phospholipids in very large HDL** | -0.02 (-0.04 to 0.01) | 0.406 | -0.02 (-0.05 to NA) | 0.332 | -0.03 (-0.05 to 0.98) | 0.184 |
| **Concentration of HDL particles** | -0.03 (-0.07 to 0.01) | 0.437 | -0.02 (-0.06 to NA) | 0.683 | -0.02 (-0.06 to 0.62) | 0.659 |
| **Total lipids in small HDL** | -0.03 (-0.08 to 0.01) | 0.437 | -0.01 (-0.06 to NA) | 0.768 | -0.02 (-0.07 to 0.55) | 0.674 |
| **HDL cholesterol** | -0.02 (-0.05 to 0.01) | 0.442 | -0.02 (-0.06 to NA) | 0.520 | -0.02 (-0.05 to 0.85) | 0.514 |
| **Cholesteryl esters in VLDL** | -0.02 (-0.06 to 0.01) | 0.442 | -0.01 (-0.05 to NA) | 0.753 | -0.02 (-0.06 to 0.8) | 0.567 |
| **Concentration of very large HDL particles** | -0.01 (-0.03 to 0.01) | 0.442 | -0.02 (-0.04 to NA) | 0.418 | -0.02 (-0.04 to 0.97) | 0.201 |
| **Glycine** | -0.02 (-0.06 to 0.01) | 0.458 | -0.04 (-0.07 to NA) | 0.332 | -0.03 (-0.07 to 0.74) | 0.419 |
| **Free cholesterol in small HDL** | -0.03 (-0.08 to 0.02) | 0.458 | -0.01 (-0.06 to NA) | 0.753 | -0.02 (-0.06 to 0.58) | 0.731 |
| **Total concentration of lipoprotein particles** | -0.03 (-0.07 to 0.01) | 0.458 | -0.01 (-0.05 to NA) | 0.753 | -0.01 (-0.06 to 0.62) | 0.723 |
| **Cholesterol in chylomicrons and extremely large VLDL** | -0.02 (-0.05 to 0.01) | 0.458 | -0.02 (-0.05 to NA) | 0.625 | -0.02 (-0.06 to 0.52) | 0.419 |
| **Cholesteryl esters in chylomicrons and extremely large VLDL** | -0.02 (-0.05 to 0.01) | 0.458 | -0.02 (-0.05 to NA) | 0.625 | -0.02 (-0.06 to 0.51) | 0.425 |
| **Cholesterol in large HDL** | -0.02 (-0.04 to 0.01) | 0.473 | -0.02 (-0.05 to NA) | 0.452 | -0.02 (-0.05 to 1.09) | 0.378 |
| **Triglycerides in medium VLDL** | 0.02 (-0.01 to 0.06) | 0.483 | 0.03 (-0.01 to NA) | 0.471 | 0.02 (-0.02 to 0.48) | 0.506 |
| **Free cholesterol in chylomicrons and extremely large VLDL** | -0.02 (-0.05 to 0.01) | 0.483 | -0.02 (-0.05 to NA) | 0.625 | -0.02 (-0.06 to 0.53) | 0.419 |
| **Cholesteryl esters in HDL** | -0.02 (-0.05 to 0.01) | 0.492 | -0.02 (-0.06 to NA) | 0.608 | -0.02 (-0.05 to 0.84) | 0.567 |
| **Total lipids in very large HDL** | -0.01 (-0.03 to 0.01) | 0.492 | -0.02 (-0.04 to NA) | 0.418 | -0.02 (-0.04 to 0.98) | 0.251 |
| **Cholesteryl esters in large HDL** | -0.02 (-0.04 to 0.01) | 0.520 | -0.02 (-0.05 to NA) | 0.482 | -0.02 (-0.05 to 1.1) | 0.419 |
| **Free cholesterol in medium LDL** | 0.03 (-0.02 to 0.08) | 0.520 | 0.05 (0 to NA) | 0.282 | 0.05 (0 to 0.62) | 0.251 |
| **Total lipids in large VLDL** | 0.02 (-0.01 to 0.05) | 0.534 | 0.02 (-0.01 to NA) | 0.482 | 0.02 (-0.02 to 0.48) | 0.555 |
| **Phospholipids in IDL** | -0.02 (-0.06 to 0.02) | 0.547 | -0.01 (-0.05 to NA) | 0.889 | -0.01 (-0.06 to 0.86) | 0.739 |
| **Saturated fatty acids** | -0.02 (-0.05 to 0.01) | 0.554 | -0.01 (-0.04 to NA) | 0.832 | -0.02 (-0.05 to 0.69) | 0.578 |
| **Triglycerides in very large VLDL** | 0.02 (-0.02 to 0.05) | 0.554 | 0.02 (-0.02 to NA) | 0.520 | 0.01 (-0.02 to 0.44) | 0.659 |
| **Omega-3 fatty acids** | -0.02 (-0.07 to 0.02) | 0.567 | -0.02 (-0.07 to NA) | 0.674 | -0.02 (-0.06 to 0.53) | 0.740 |
| **VLDL cholesterol** | -0.02 (-0.05 to 0.02) | 0.579 | -0.01 (-0.05 to NA) | 0.839 | -0.02 (-0.06 to 0.79) | 0.674 |
| **Total lipids in IDL** | -0.02 (-0.06 to 0.02) | 0.585 | 0 (-0.05 to NA) | 0.924 | -0.01 (-0.06 to 0.87) | 0.785 |
| **Sphingomyelins** | -0.02 (-0.06 to 0.02) | 0.585 | 0 (-0.05 to NA) | 0.904 | -0.01 (-0.05 to 0.72) | 0.884 |
| **Citrate** | 0.03 (-0.03 to 0.08) | 0.591 | 0.02 (-0.03 to NA) | 0.625 | 0.03 (-0.03 to 0.65) | 0.561 |
| **Phospholipids in chylomicrons and extremely large VLDL** | -0.02 (-0.05 to 0.02) | 0.591 | -0.01 (-0.05 to NA) | 0.700 | -0.02 (-0.06 to 0.51) | 0.506 |
| **Concentration of VLDL particles** | -0.02 (-0.05 to 0.02) | 0.638 | -0.01 (-0.05 to NA) | 0.832 | -0.02 (-0.06 to 0.77) | 0.671 |
| **Remnant cholesterol (non-HDL, non-LDL -cholesterol)** | -0.02 (-0.06 to 0.02) | 0.659 | 0 (-0.05 to NA) | 0.958 | -0.01 (-0.05 to 0.83) | 0.812 |
| **Total lipids in small LDL** | 0.02 (-0.02 to 0.06) | 0.659 | 0.03 (-0.01 to NA) | 0.417 | 0.03 (-0.01 to 0.64) | 0.410 |
| **Free cholesterol in IDL** | -0.02 (-0.06 to 0.02) | 0.670 | 0 (-0.05 to NA) | 1.000 | -0.01 (-0.05 to 0.88) | 0.884 |
| **Cholesteryl esters in large LDL** | 0.02 (-0.03 to 0.07) | 0.680 | 0.04 (-0.01 to NA) | 0.400 | 0.04 (-0.01 to 0.68) | 0.402 |
| **Histidine** | -0.02 (-0.07 to 0.03) | 0.690 | -0.02 (-0.07 to NA) | 0.608 | -0.01 (-0.06 to 0.61) | 0.785 |
| **Total lipids in small VLDL** | -0.02 (-0.05 to 0.02) | 0.693 | -0.01 (-0.05 to NA) | 0.833 | -0.01 (-0.05 to 0.68) | 0.762 |
| **Triglycerides in VLDL** | 0.01 (-0.02 to 0.05) | 0.693 | 0.02 (-0.02 to NA) | 0.625 | 0.01 (-0.02 to 0.47) | 0.740 |
| **Free cholesterol in LDL** | 0.02 (-0.03 to 0.07) | 0.736 | 0.04 (-0.01 to NA) | 0.417 | 0.04 (-0.01 to 0.69) | 0.410 |
| **3-Hydroxybutyrate** | -0.02 (-0.08 to 0.04) | 0.744 | -0.02 (-0.08 to NA) | 0.700 | -0.02 (-0.08 to 0.56) | 0.686 |
| **Cholesterol in large LDL** | 0.02 (-0.03 to 0.07) | 0.744 | 0.04 (-0.01 to NA) | 0.418 | 0.04 (-0.01 to 0.69) | 0.419 |
| **LDL cholesterol** | 0.02 (-0.03 to 0.06) | 0.744 | 0.04 (-0.01 to NA) | 0.418 | 0.04 (-0.01 to 0.67) | 0.419 |
| **Cholesteryl esters in LDL** | 0.02 (-0.03 to 0.06) | 0.744 | 0.04 (-0.01 to NA) | 0.418 | 0.03 (-0.01 to 0.66) | 0.425 |
| **Phospholipids in medium LDL** | 0.02 (-0.03 to 0.06) | 0.744 | 0.04 (-0.01 to NA) | 0.418 | 0.03 (-0.01 to 0.59) | 0.419 |
| **Cholesterol in small LDL** | 0.02 (-0.03 to 0.06) | 0.744 | 0.03 (-0.01 to NA) | 0.418 | 0.03 (-0.01 to 0.64) | 0.419 |
| **Concentration of small LDL particles** | 0.01 (-0.02 to 0.05) | 0.744 | 0.02 (-0.02 to NA) | 0.520 | 0.02 (-0.02 to 0.75) | 0.555 |
| **Phospholipids in small VLDL** | -0.01 (-0.05 to 0.02) | 0.752 | 0 (-0.04 to NA) | 0.963 | -0.01 (-0.05 to 0.72) | 0.884 |
| **Total lipids in lipoprotein particles** | -0.01 (-0.05 to 0.03) | 0.754 | 0 (-0.04 to NA) | 0.967 | -0.01 (-0.05 to 0.83) | 0.884 |
| **Total lipids in very large VLDL** | 0.01 (-0.02 to 0.04) | 0.754 | 0.02 (-0.02 to NA) | 0.625 | 0.01 (-0.03 to 0.47) | 0.805 |
| **Concentration of large VLDL particles** | 0.01 (-0.02 to 0.04) | 0.762 | 0.02 (-0.02 to NA) | 0.625 | 0.01 (-0.03 to 0.49) | 0.776 |
| **Cholesterol in medium LDL** | 0.01 (-0.03 to 0.06) | 0.770 | 0.03 (-0.01 to NA) | 0.439 | 0.03 (-0.02 to 0.62) | 0.459 |
| **Cholesterol in IDL** | -0.01 (-0.06 to 0.03) | 0.771 | 0.01 (-0.04 to NA) | 0.894 | 0 (-0.05 to 0.89) | 0.973 |
| **Total lipids in medium VLDL** | 0.01 (-0.03 to 0.05) | 0.771 | 0.02 (-0.02 to NA) | 0.580 | 0.02 (-0.03 to 0.71) | 0.659 |
| **Phospholipids in VLDL** | -0.01 (-0.04 to 0.02) | 0.779 | 0 (-0.04 to NA) | 0.958 | -0.01 (-0.05 to 0.69) | 0.786 |
| **Cholesteryl esters in very large HDL** | -0.01 (-0.03 to 0.01) | 0.801 | -0.01 (-0.03 to NA) | 0.625 | -0.01 (-0.04 to 0.98) | 0.523 |
| **Free cholesterol in VLDL** | -0.01 (-0.05 to 0.03) | 0.802 | 0 (-0.04 to NA) | 1.000 | -0.01 (-0.05 to 0.74) | 0.832 |
| **Cholesterol in very large HDL** | -0.01 (-0.03 to 0.01) | 0.802 | -0.01 (-0.03 to NA) | 0.625 | -0.01 (-0.04 to 0.97) | 0.516 |
| **Creatinine** | -0.01 (-0.03 to 0.02) | 0.809 | -0.01 (-0.04 to NA) | 0.608 | -0.01 (-0.03 to 0.41) | 0.832 |
| **Cholesteryl esters in IDL** | -0.01 (-0.06 to 0.03) | 0.816 | 0.01 (-0.04 to NA) | 0.844 | 0 (-0.05 to 0.87) | 0.990 |
| **Clinical LDL cholesterol** | 0.01 (-0.04 to 0.06) | 0.819 | 0.03 (-0.02 to NA) | 0.482 | 0.03 (-0.02 to 0.7) | 0.506 |
| **Concentration of large LDL particles** | 0.01 (-0.03 to 0.05) | 0.819 | 0.02 (-0.02 to NA) | 0.547 | 0.02 (-0.02 to 0.71) | 0.555 |
| **Total lipids in LDL** | 0.01 (-0.03 to 0.05) | 0.819 | 0.03 (-0.02 to NA) | 0.486 | 0.03 (-0.02 to 0.65) | 0.514 |
| **Concentration of LDL particles** | 0.01 (-0.03 to 0.05) | 0.819 | 0.02 (-0.02 to NA) | 0.547 | 0.02 (-0.02 to 0.71) | 0.555 |
| **Phospholipids in LDL** | 0.01 (-0.03 to 0.06) | 0.819 | 0.03 (-0.02 to NA) | 0.486 | 0.03 (-0.02 to 0.65) | 0.506 |
| **Average diameter for LDL particles** | -0.01 (-0.07 to 0.04) | 0.819 | 0 (-0.06 to NA) | 0.940 | 0 (-0.06 to 0.88) | 0.982 |
| **Total lipids in medium LDL** | 0.01 (-0.03 to 0.05) | 0.819 | 0.03 (-0.02 to NA) | 0.482 | 0.03 (-0.02 to 0.61) | 0.506 |
| **Concentration of small HDL particles** | -0.01 (-0.06 to 0.04) | 0.819 | 0.01 (-0.04 to NA) | 0.819 | 0.01 (-0.04 to 0.56) | 0.884 |
| **Free cholesterol in small VLDL** | -0.01 (-0.05 to 0.03) | 0.819 | 0 (-0.04 to NA) | 0.910 | 0 (-0.04 to 0.72) | 0.990 |
| **Concentration of small VLDL particles** | -0.01 (-0.05 to 0.03) | 0.819 | 0 (-0.05 to NA) | 0.958 | -0.01 (-0.05 to 0.67) | 0.884 |
| **Total free cholesterol** | -0.01 (-0.05 to 0.03) | 0.819 | 0.01 (-0.04 to NA) | 0.884 | 0 (-0.04 to 0.84) | 0.990 |
| **Free cholesterol in very large HDL** | 0 (-0.03 to 0.02) | 0.819 | -0.01 (-0.03 to NA) | 0.659 | -0.01 (-0.04 to 0.93) | 0.523 |
| **Concentration of chylomicrons and extremely large VLDL particles** | -0.01 (-0.04 to 0.03) | 0.832 | 0 (-0.04 to NA) | 0.904 | -0.01 (-0.05 to 0.51) | 0.686 |
| **Triglycerides in chylomicrons and extremely large VLDL** | 0.01 (-0.03 to 0.04) | 0.832 | 0.01 (-0.03 to NA) | 0.775 | 0 (-0.03 to 0.53) | 0.990 |
| **Cholesteryl esters in medium LDL** | 0.01 (-0.03 to 0.05) | 0.838 | 0.03 (-0.02 to NA) | 0.516 | 0.02 (-0.02 to 0.62) | 0.555 |
| **Free cholesterol in large LDL** | 0.01 (-0.04 to 0.06) | 0.838 | 0.03 (-0.02 to NA) | 0.501 | 0.03 (-0.02 to 0.74) | 0.516 |
| **Cholesteryl esters in medium VLDL** | -0.01 (-0.05 to 0.04) | 0.838 | 0.01 (-0.04 to NA) | 0.833 | 0 (-0.04 to 0.82) | 0.926 |
| **Total lipids in large LDL** | 0.01 (-0.04 to 0.06) | 0.844 | 0.03 (-0.02 to NA) | 0.518 | 0.03 (-0.02 to 0.68) | 0.530 |
| **Total cholesterol** | -0.01 (-0.05 to 0.04) | 0.850 | 0.01 (-0.04 to NA) | 0.813 | 0.01 (-0.04 to 0.82) | 0.889 |
| **Concentration of very large VLDL particles** | 0.01 (-0.03 to 0.04) | 0.856 | 0.01 (-0.02 to NA) | 0.742 | 0 (-0.03 to 0.48) | 0.910 |
| **Phospholipids in large VLDL** | 0.01 (-0.03 to 0.04) | 0.861 | 0.01 (-0.03 to NA) | 0.734 | 0.01 (-0.03 to 0.51) | 0.889 |
| **Concentration of medium LDL particles** | 0.01 (-0.03 to 0.04) | 0.861 | 0.02 (-0.02 to NA) | 0.608 | 0.02 (-0.02 to 0.65) | 0.662 |
| **Cholesterol in small HDL** | -0.01 (-0.06 to 0.04) | 0.861 | 0.01 (-0.04 to NA) | 0.768 | 0.01 (-0.04 to 0.56) | 0.832 |
| **Cholesteryl esters in large VLDL** | -0.01 (-0.05 to 0.03) | 0.867 | 0 (-0.04 to NA) | 0.919 | 0 (-0.05 to 0.75) | 0.942 |
| **Free cholesterol in large VLDL** | 0 (-0.03 to 0.04) | 0.867 | 0.01 (-0.02 to NA) | 0.719 | 0.01 (-0.03 to 0.53) | 0.884 |
| **Total esterified cholesterol** | -0.01 (-0.05 to 0.04) | 0.867 | 0.01 (-0.03 to NA) | 0.768 | 0.01 (-0.04 to 0.78) | 0.874 |
| **Docosahexaenoic acid** | 0 (-0.04 to 0.03) | 0.900 | -0.01 (-0.05 to NA) | 0.833 | 0 (-0.04 to 0.62) | 0.973 |
| **Concentration of medium VLDL particles** | 0.01 (-0.03 to 0.04) | 0.900 | 0.02 (-0.03 to NA) | 0.659 | 0.01 (-0.03 to 0.77) | 0.776 |
| **Phospholipids in very large VLDL** | 0 (-0.03 to 0.03) | 0.905 | 0.01 (-0.03 to NA) | 0.768 | 0 (-0.03 to 0.5) | 0.978 |
| **Total lipids in chylomicrons and extremely large VLDL** | 0 (-0.03 to 0.03) | 0.910 | 0 (-0.04 to NA) | 1.000 | -0.01 (-0.04 to 0.52) | 0.786 |
| **Cholesterol in medium VLDL** | 0 (-0.05 to 0.04) | 0.915 | 0.01 (-0.04 to NA) | 0.782 | 0.01 (-0.04 to 0.82) | 0.884 |
| **Cholesteryl esters in small LDL** | 0 (-0.03 to 0.04) | 0.915 | 0.02 (-0.02 to NA) | 0.608 | 0.02 (-0.02 to 0.64) | 0.659 |
| **Cholesteryl esters in very large VLDL** | 0 (-0.04 to 0.03) | 0.915 | 0.01 (-0.03 to NA) | 0.832 | 0 (-0.04 to 0.67) | 0.990 |
| **Triglycerides in small VLDL** | 0 (-0.04 to 0.04) | 0.930 | 0 (-0.05 to NA) | 0.919 | -0.01 (-0.05 to 0.57) | 0.889 |
| **Apolipoprotein B** | 0 (-0.04 to 0.04) | 0.976 | 0.01 (-0.03 to NA) | 0.734 | 0.01 (-0.03 to 0.73) | 0.832 |
| **Phospholipids in medium VLDL** | 0 (-0.04 to 0.04) | 0.976 | 0.02 (-0.03 to NA) | 0.711 | 0.01 (-0.03 to 0.78) | 0.832 |
| **Cholesterol in very large VLDL** | 0 (-0.03 to 0.03) | 0.976 | 0.01 (-0.03 to NA) | 0.832 | 0 (-0.04 to 0.59) | 0.990 |
| **Phospholipids in large LDL** | 0 (-0.05 to 0.05) | 0.979 | 0.02 (-0.03 to NA) | 0.625 | 0.02 (-0.03 to 0.69) | 0.662 |
| **Free cholesterol in medium VLDL** | 0 (-0.04 to 0.04) | 0.979 | 0.02 (-0.03 to NA) | 0.711 | 0.01 (-0.03 to 0.79) | 0.831 |
| **Total triglycerides** | 0 (-0.03 to 0.03) | 0.979 | 0 (-0.03 to NA) | 0.958 | 0 (-0.04 to 0.51) | 0.894 |
| **Total cholesterol minus HDL-C** | 0 (-0.04 to 0.04) | 0.996 | 0.02 (-0.03 to NA) | 0.665 | 0.01 (-0.03 to 0.74) | 0.784 |
| **Cholesteryl esters in small HDL** | 0 (-0.05 to 0.04) | 0.996 | 0.02 (-0.03 to NA) | 0.655 | 0.02 (-0.03 to 0.63) | 0.686 |
| **Total lipids in VLDL** | 0 (-0.03 to 0.03) | 0.996 | 0.01 (-0.03 to NA) | 0.832 | 0 (-0.04 to 0.6) | 0.990 |
| **Free cholesterol in very large VLDL** | 0 (-0.03 to 0.03) | 0.996 | 0.01 (-0.03 to NA) | 0.833 | 0 (-0.03 to 0.53) | 0.986 |
| **Cholesterol in large VLDL** | 0 (-0.04 to 0.04) | 1.000 | 0.01 (-0.03 to NA) | 0.826 | 0 (-0.04 to 0.64) | 0.990 |

Basic model is adjusted for adjusted for age, sex, mosques, and day of the second measurement; model i is adjusted for variables in the basic model, waist circumference, free-fat mass and body mass index on top of variable; model ii is adjusted for variables in the basic model, total body water and fat percentage.

**Supplemental Table 4:** Associations between metabolic scores and chronic diseases in UK Biobank adjusted for age and sex.

| **Disease** | **OR** | **95% CI** | **P-value** |
| --- | --- | --- | --- |
| **Diabetes** | 1.25 | 1.23 to 1.26 | <0.001 |
| **Hypertension** | 1.06 | 1.04 to 1.08 | <0.001 |
| **coronary heart disease** | 2.27 | 2.15 to 2.39 | <0.001 |
| **Renal failure** | 2.72 | 2.63 to 2.80 | <0.001 |
| **Colorectal cancer** | 3 | 2.07 to 4.32 | <0.001 |
| **Breast cancer** | 2.78 | 1.53 to 5.07 | <0.001 |
| **Lung Cancer** | 2.95 | 2.65 to 3.31 | <0.001 |

**Supplemental Table 5:** Changes in metabolic scores after Ramadan in LORANS.

| **Disease** | **Change after Ramadan* (% compared to before Ramadan)** | **95% CI** | **p.value** |
| --- | --- | --- | --- |
| **Diabetes** | 1.08 (1.9) | -2.40 to 4.58 | 0.545 |
| **Hypertension** | -0. 75 (-2.1) | -3.35 to 1.86 | 0. 574 |
| **coronary heart disease** | -0. 71 (-2) | -3.33 to 1.91 | 0.594 |
| **Renal failure** | -0. 72 (-2.1) | -3.34 to 1.89 | 0.586 |
| **Colorectal cancer** | -1.09 (-2.4) | -1.69 to -0. 50 | <0.001 |
| **Breast cancer** | -0.48 (-1.1) | -0. 81 to -0.15 | 0.006 |
| **Lung cancer** | -4.74 (-9.6) | -6.56 to -2.91 | <0.001 |

* Change is scaled between 0 to 100

**Supplemental Table 6:** Sensitivity analysis to test whether changes observed after Ramadan fasting in metabolic scores of colorectal cancer and lung cancer were driven by glycoprotein acetyls.

| **Disease** | **Original score (including GlycA)/ without GlycA** | **metabolites contributing to metabolic score** | **in LORANS** | | |
| --- | --- | --- | --- | --- | --- |
|  |  |  | **Change after Ramadan* (% compared to before Ramadan)** | **95% CI** | **p.value** |
| **Colorectal cancer** | Original score (including GlycA) | Free Cholesterol in IDL  Glycoprotein acetyls | -1.09 (-2.4) | -1.69 to -0.50 | <0.001 |
| **Colorectal cancer** | without GlycA | Free Cholesterol in IDL | 0.19 (0.42) | -0. 24 to 0.62 | 0.387 |
| **Lung cancer** | Original score (including GlycA) | Glycoprotein acetyls  Linoleic acid  Omega 3 fatty acids  Citrate  Degree of unsaturation  Alanine  Cholesteryl esters in very large HDL  Lactate  Β_hydroxybutyrate | -4.74 (-9.62) | -6.56 to -2.91 | <0.001 |
| **Lung cancer** | without GlycA | Glycoprotein acetyls  Linoleic acid  Omega 3 fatty acids  Citrate  Degree of unsaturation  Alanine  Cholesteryl esters in very large HDL  Lactate  β_hydroxybutyrate | -2.05 (-4.88) | -4.19 to 0.08 | 0.062 |

* Change is scaled between 0 to 100; GlycA, Glycoprotein acetyls.

**Supplemental table 7:** Comparison of two metabolic scores of lung cancer (with/without adjusting for smoking).

| **Method** | **Adjustment in the initial univariate model** | **Number of metabolites highly correlated with lung Cancer using univariate model** | **Number of metabolites surviving LASSO regression** | **Number of metabolites significantly correlated with lung Cancer using multivariate model** | **Metabolites used to create the metabolic score** | **Change in the metabolic score after Ramadan in LORANS participants* (%)** | **p-value of change after Ramadan in LORANS** |
| --- | --- | --- | --- | --- | --- | --- | --- |
| 1 | None | 88 | 11 | 9 | Glycoprotein acetyls  Linoleic acid  Omega 3 fatty acids  Citrate  Degree of unsaturation  Alanine  Cholesteryl esters in very large HDL  Lactate  β_hydroxybutyrate | -4.74 (-9.62) | <0.001 |
| 2 | Smoking intensity | 12 | 8 | 6 | Glycoprotein acetyls  Alanine  β_hydroxybutyrate  Degree of Unsaturation  Lactate  Polyunsaturated Fatty Acids | -2.95 (-5.69) | 0.001 |

* change is scaled between 0 to 100

**Supplemental Table 8:** change in smoking metabolic score after Ramadan fasting amongst LORANS participants.

| **Number of metabolites significantly correlated with lung Cancer using multivariate model** | **Metabolites used to create the metabolic score** | **Change in the metabolic score after Ramadan in LORANS participants* (%)** | **p-value of change after Ramadan in LORANS** |
| --- | --- | --- | --- |
| 9 | Linoleic Acid  Degree of Unsaturation  Glycoprotein Acetyls  Citrate  Cholesteryl Esters in Chylomicrons and Extremely Large VLDL  Monounsaturated Fatty Acids  Glutamine  Lactate  Valine  Triglycerides in Large HDL  Glycine  Triglycerides in Large LDL  Acetone  Acetate  Albumin  NMR Creatinine  Alanine  Acetoacetate | -0. 98 (-2.13) | 0.570 |

* change is scaled between 0 to 100
